# Supplementary material for: Multifaceted consequences of visual distraction during natural behaviour
Source: Commun Psychol. 2024 May 27;2:49. doi: 10.1038/s44271-024-00099-0 (PMC11129948; doi:10.1038/s44271-024-00099-0)
Supplement: Supplementary file 2 — Supplementary Information [file 44271_2024_99_MOESM2_ESM.pdf]

Supplementary Notes 1

Participants encoded more individual target objects from the Model when movement effort was high (β =- 0.16, *SE* = 0.01, *z* =-10.97, *p* < 0.001, $CI_{95\%}$ = [-0.19,-0.13]; *Supplementary Figure 1A*). Distraction did not interfere with the number of targets encoded (β = 0.006, *SE* = 0.004, *z* = 1.62, *p* = 0.11, $CI_{95\%}$ = [-0.001,0.01]), and did not interact with movement effort (β = 0.003, *SE* = 0.004, *z* = 0.69, *p* = 0.49, $CI_{95\%}$ = [-0.01,0.01]).

To investigate whether encoding time predicted subsequent memory usage, we included Model viewing time as an additive predictor to the generalized linear mixed model predicting WM usage by distraction and movement effort (see Methods for details). Encoding time positively predicted WM usage (β = 0.05, *SE* = 0.003, *z* = 15.15, *p* < 0.001, $CI_{95\%}$ = [0.05,0.06]; *Supplementary Figure 1B*): Participants relied on memory more following longer encoding periods.

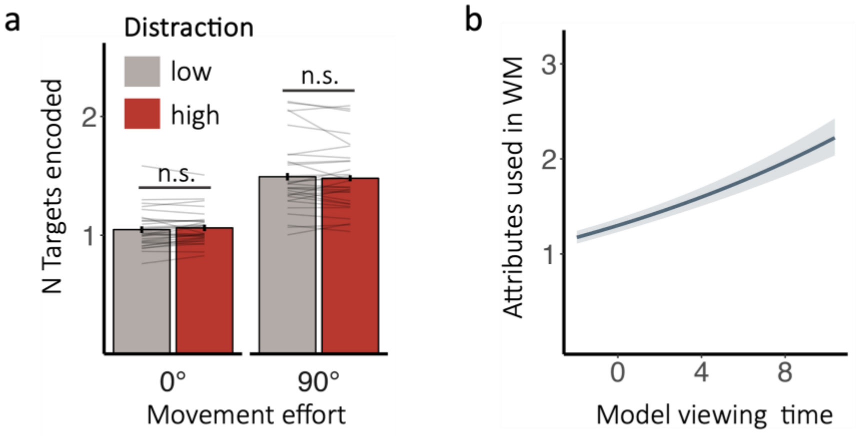

Supplementary Figure 1. *Participants encode more information when movement effort is high*. **a)** Participants encoded more individual target objects from the Model when movement effort was high, but distraction did not interfere with the number of targets encoded. **b)** Model viewing time (centered) predicted subsequent memory usage. Participants relied on memory more following longer encoding periods. Error bars depict standard error of the mean (*N* = 30). Lines show individual participant data. The symbols *, **, and *** in the figure denote statistical significance with p-values less than 0.05, 0.01, and 0.001, respectively.

Supplementary Notes 2

Given the extended nature of the task, we additionally conducted post-hoc control analyses into potential temporal effects in the core subcomponent metrics over the course of one trial (i.e., trial progress). Overall, trial progress did affect behaviour across multiple subcomponents, highlighting that temporal effects are an important dimension in extended tasks. Critically, including trial progress as a predictor did not affect our conclusions regarding the effects of visual distraction, movement effort, or their interaction.

Analysis. To investigate potential temporal changes over the course of one trial, we included trial progress (i.e., number of targets already placed at given point in trial) as an additive continuous predictor (both as a linear and quadratic effect) to the final models of metrics in different subcomponents of behaviour (i.e., model viewing time, attributes used in memory, and search time; see *Supplementary Figure 2*). Given the presence of both categorical (i.e., distraction and movement effort) and continuous predictors, we report β coefficients and their 95% confidence intervals for both LMMs and GLMMs.

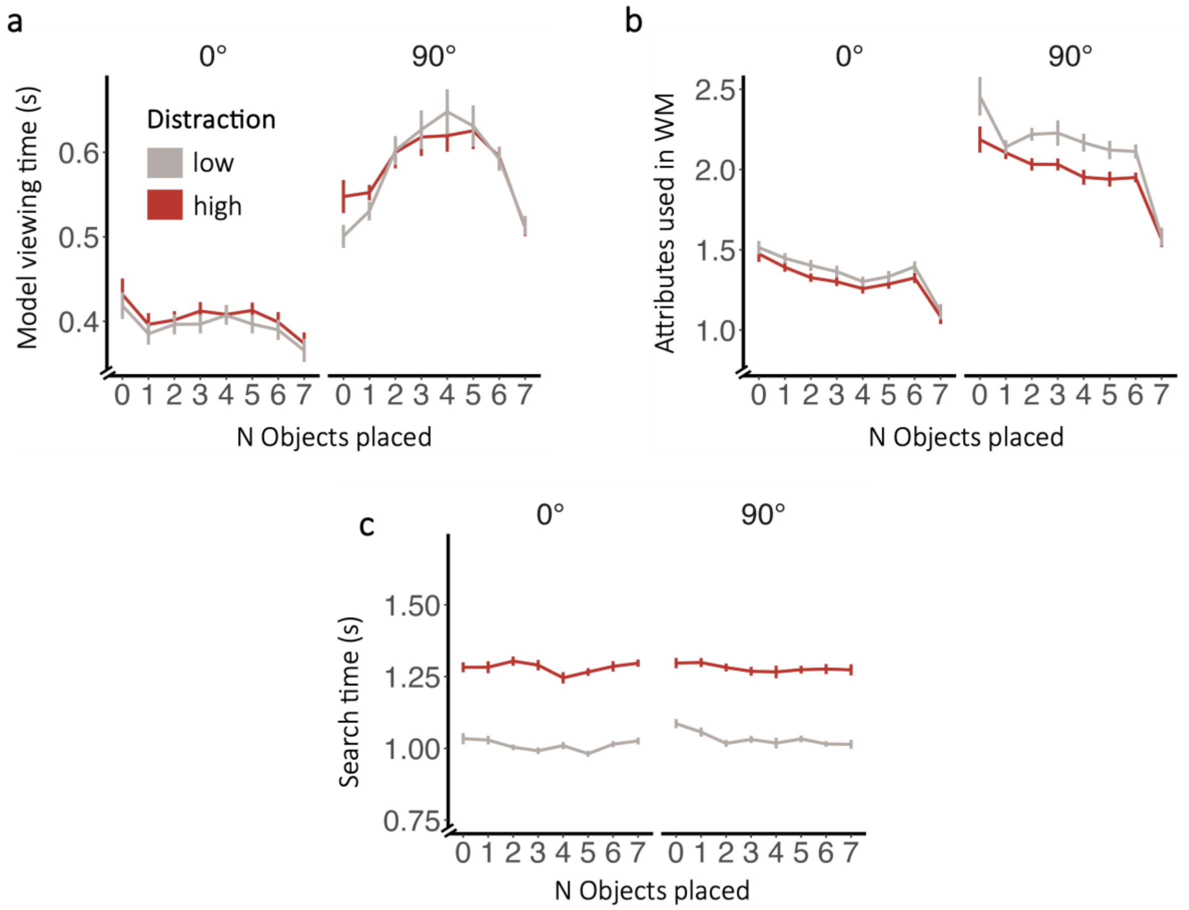

Supplementary Figure 2. *Temporal task effects on metrics from different subcomponents*. Trial progress (as indexed by the number of target objects already placed at a given time within the trial) affected metrics across multiple subcomponents (i.e., **a)** Encoding as indexed by model viewing times, **b)** WM usage as indexed by Attributes used in WM, and **c)** Visual search as indexed by search time). *Error bars depict standard error of the mean* (N *= 30).*

Results. Specifically, trial progress predicted model viewing time, including both a linear (β =-3.50, SE = 0.36, t =-9.84, p < 0.001, $CI_{95\%}$ = [-4.19,-2.80]) and quadratic (β =-4.83, SE = 0.35, t =-13.63, p < 0.001, $CI_{95\%}$ = [-5.53,-4.14]) effect (see *Supplementary Figure 2A*). However, even after the inclusion of trial progress as a predictor, we again observed no meaningful difference in model viewing times between distraction conditions (β = 0.01, SE = 0.03, t = 1.88, p = 0.07, $CI_{95\%}$ = [-0.0003,0.01]). Additionally, movement effort still predicted model viewing time (β =-0.19, SE = 0.02, t =-11.43, p < 0.001, $CI_{95\%}$ = [-0.22,-0.15]). That is, participants still encoded from the model for longer when movement effort was high. Further, the effect of distraction on model viewing time was weakly but reliably moderated by movement effort (β = 0.01, SE = 0.003, t = 2.39, p = 0.02, $CI_{95\%}$ = [0.001,0.01]).

Trial progress also predicted attributes used in WM, including both a linear (β =-16.31, SE = 0.71, z =-22.89, p < 0.001, $CI_{95\%}$ = [-17.70,-14.91]) and quadratic (β =-3.09, SE = 0.64, t =-4.85, p < 0.001, $CI_{95\%}$ = [-4.33,-1.84]) effect (see *Supplementary Figure 2B).* After including trial progress as a predictor, high distraction again decreased (β =-0.03, SE = 0.004, z =-7.04, p < 0.001, $CI_{95\%}$ = [-0.03,-0.019]) and high movement effort increased (β =-0.21, SE = 0.01, z =-17.99, p < 0.001, $CI_{95\%}$ = [-0.23,-0.18]) the number of attributes used in WM. Distraction also interacted with movement effort (β = 0.01, SE = 0.003, z = 2.32, p = 0.02, $CI_{95\%}$ = [0.001,0.01]).

We additionally found a linear (β =-2.79, SE = 0.43, t =-6.43, p < 0.001, $CI_{95\%}$ = [-3.64,-1.93]) and quadratic (β = 2.15, SE = 0.43, t = 4.95, p < 0.001, $CI_{95\%}$ = [-0.23,-0.18]) effect of trial progress on search time (see *Supplementary Figure 2C*). After including trial progress as a predictor, high distraction still slowed down visual search (β =0.1, SE = 0.004, t = 26.15, p < 0.001, $CI_{95\%}$ = [0.09,0.11]) but we observe no difference in search time between movement effort conditions, β < 0.001, SE = 0.004, t = 0.11, p = 0.92, $CI_{95\%}$ = [-0.01,0.01]). Distraction and movement effort also again interacted (β = 0.01, SE = 0.003, t = 2.32, p = 0.03, $CI_{95\%}$ = [0.001,0.01]).

Supplementary Notes 3

The flexibility in self-structuring behaviour during the task allowed for potential strategy changes. That is, behavioural sequences could have a different structure, even if participants are using the same number of attributes in WM (see *Supplementary Figure 3*). For example, using two attributes in WM could be the result of first searching for and picking up a target object (i.e., "search and pick-up first" sequences, *Supplementary Figure 3A*) and then directly placing this object (i.e., location attribute used; one entire object copied; sequence type S2 in *Supplementary Figure 3B*). At the same time, participants could have used two attributes in WM by first placing an already picked-up object (i.e., "Placement first" sequence; participant encoded while holding an object in hand *Supplementary Figure 3A*), before continuing to search for and pick-up another object (i.e., the two attributes are split between two objects; sequence type P2 in *Supplementary Figure 3B*). Here, we further broke down overall WM usage to test for any changes related to the structure of behavioural sequences.

Analysis. We first determined different sequence types by dividing behavioural sequences according to the number of attributes used within them, as well whether they started with search and pick-up (i.e., "search and pick-up first" sequences, *Supplementary Figure 3A*) or placement (i.e., "Placement first" sequence, *Supplementary Figure 3A*). We then computed the probability of different sequence types. Differences in means between distraction conditions were analysed nested in movement effort conditions, using paired pairwise t-tests as well as paired Bayesian t-tests. All pairwise comparisons are reported in *Supplementary Table 1* below.

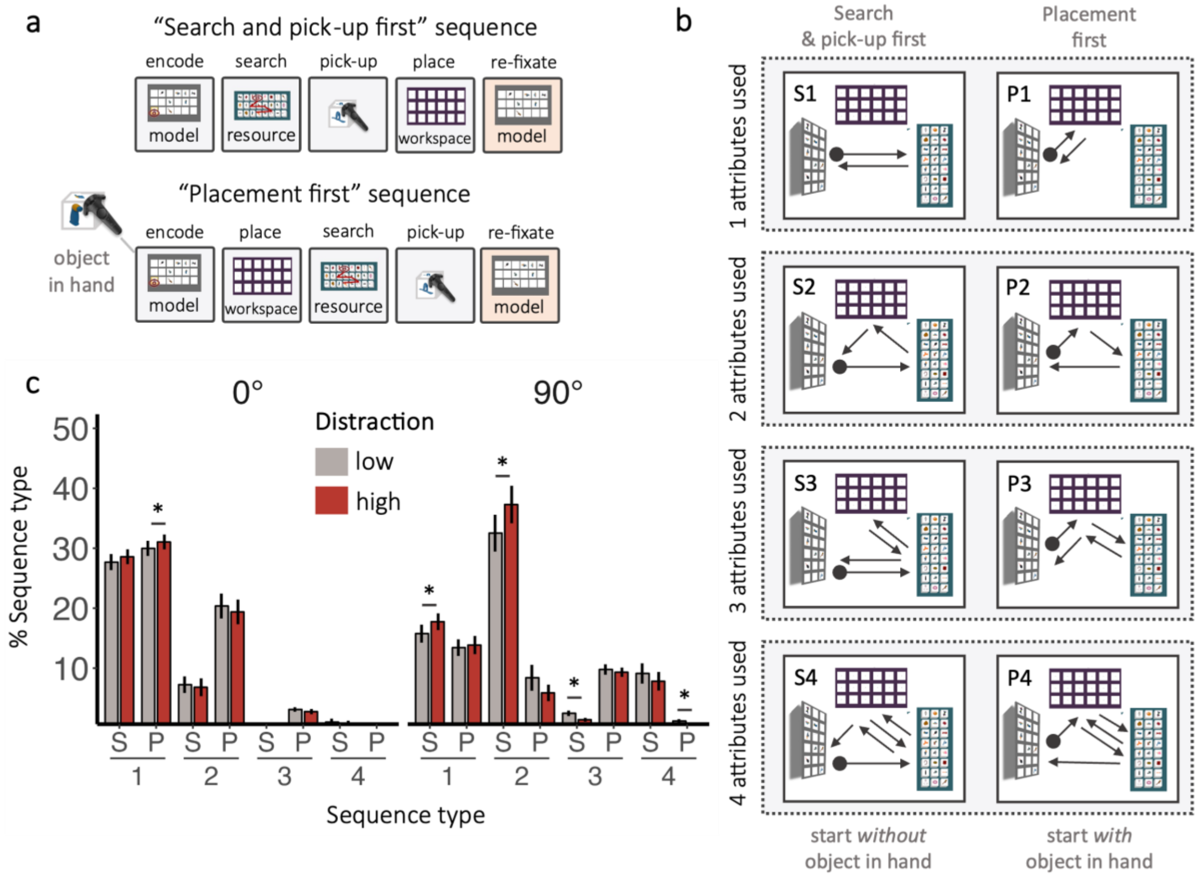

Supplementary Figure 3. *Flexibility in self-structuring behaviour introduces possibilities for strategy changes. a)* Behavioural sequences could have a different structure, even if participants are using the same number of attributes in WM. That is, sequences could start with search and pick-up (i.e., "Search and pick-up first"-sequences; sequence starts without object in hand) or placement right after encoding (i.e., "Placement first"-sequences;

sequence starts with object in hand). **b)** Illustration of the structure of different sequence types (in 90° movement effort condition). **c)** Probability of using different sequence types. S indicates sequences that started with search, P indicates sequences that started with placement. Error bars depict standard error of the mean (N = 30). The symbols *, **, and *** in the figure denote statistical significance with p-values less than 0.05, 0.01, and 0.001, respectively.

Results. Overall, we do not find evidence for systematic strategy shifts induced by visual distraction (see *Supplementary Figure 3* and *Supplementary Table 1*). While we do find differences between distraction conditions in some sequence types (e.g., S1 in 90° movement effort), overall patterns were unaffected and mean differences were small. Note, that the results here are a post-hoc decomposition of the differences found in the main analysis of WM usage (see **Fig. 5C**).

Supplementary Table 1
Outcomes of pairwise comparisons analysing the effect of distraction the probability of sequence types.

Measure	Comparison	*t*	*df*	p	Mean diff	Cohen's *d*	95% CI	BF_{10}
Probability Sequence Type (0° movement effort)	**S1:** high vs low distraction	-1.47	29	0.15	-0.89	-0.27	-0.63, 0.10	0.51
	**P1:** high vs low distraction	-2.55	29	0.02	-1.07	-0.47	-0.84,-0.08	2.98
	**S2:** high vs low distraction	0.98	29	0.33	0.43	0.18	-0.18, 0.54	0.30
	**P2:** high vs low distraction	0.86	29	0.40	0.98	0.16	-0.21, 0.52	0.27
	**S3:** high vs low distraction	0.84	29	0.41	0.09	0.15	-0.21, 0.51	0.27
	**P3:** high vs low distraction	1.10	29	0.28	0.36	0.20	-0.16, 0.56	0.34
	**S4:** high vs low distraction	2.16	29	0.04	0.25	0.39	0.02, 0.76	1.46
	**P4:** high vs low distraction	1.98	29	0.057	0.14	0.36	0.01, 0.73	1.10
Probability Sequence Type (90° movement effort)	**S1:** high vs low distraction	-4.08	29	< 0.001	-1.98	-0.75	-1.15,-0.33	91.53
	**P1:** high vs low distraction	-0.83	29	0.41	-0.43	-0.15	0.51, 0.21	0.27
	**S2:** high vs low distraction	-2.74	29	0.01	-4.77	-0.50	-0.88,-0.12	4.37
	**P2:** high vs low distraction	1.34	29	0.19	2.57	0.24	-0.12, 0.61	0.43
	**S3:** high vs low distraction	3.93	29	< 0.001	1.08	0.72	0.31, 1.12	64.04
	**P3:** high vs low distraction	0.89	29	0.38	0.47	0.16	-0.20, 0.52	0.28
	**S4:** high vs low distraction	1.70	29	0.10	1.30	0.31	-0.06, 0.68	0.70
	**P4:** high vs low distraction	3.21	29	0.003	0.78	0.59	0.19, 0.97	11.91

Supplementary Notes 4

In line with our a priori set analysis plans, our main analyses are focused on behavioural sequences which were initiated by participants encoding from the Model display without an object in hand (see **Fig. 6,** 70 % of sequences in 90° movement effort condition). That is, participants searched and picked up an object directly after encoding from the Model display. The first sensory-mnemonic decision in this type of behavioural sequence always was a location-related decision (i.e., decide to place the picked-up object vs. encode location from the Model first, see *Supplementary Figure 4A*).

However, given the temporally extended and unconstrained nature of the task, sequences could also start with a placement directly after encoding. Specifically, every time a participant chose to re-encode from the Model after picking-up and before placing an object, they would encode from the Model with an object already in their hand. Consequentially, the action following encoding had to be placing the grabbed object and the first sensory-mnemonic decision after this placement would be an identity-related one (i.e., pick-up another object vs. encode from the Model, see *Supplementary Figure 4B*). For completeness, we performed equivalent analyses regarding sensory-mnemonic decisions and errors in sequences during which participants encoded with an object in their hand. Note that all analyses regarding overall behaviour, encoding, search, and WM usage always include both types of sequences.

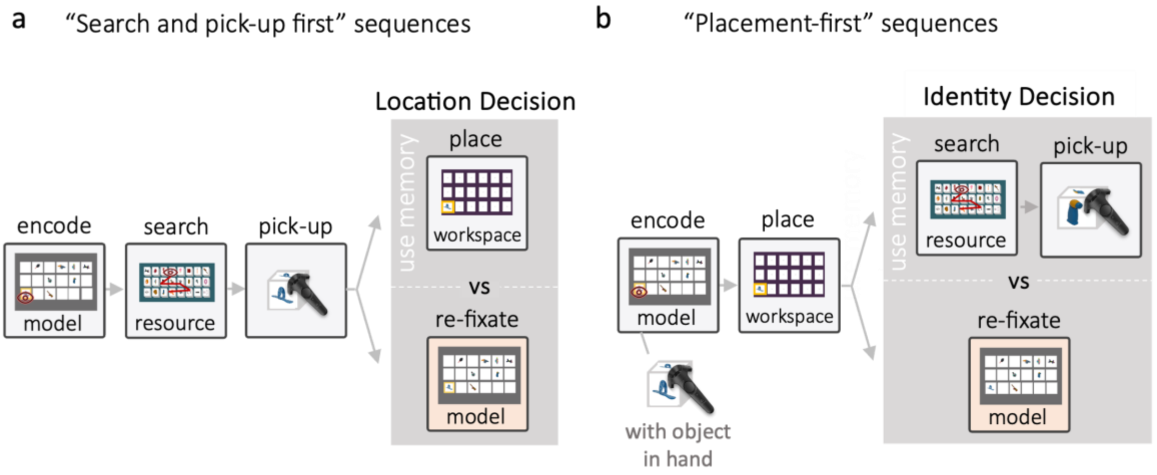

Supplementary Figure 4. *Illustration of different possible sequence types.* **a)** Sequence type included in analyses of sensory-mnemonic decisions and errors in main analyses (70 % of all sequence in 90° movement effort condition). Participants encoded without an object in their hand and the first action following encoding had to be search and pick-up. Consequentially, the sensory-mnemonic decision following pick-up had to be a location-related decision. **b)** Whenever participants chose to re-fixate the Model after pick-up, they encoded with an object already in their hand. Consequentially, sequences could also start with placement after encoding. In these cases, the first sensory-mnemonic decision was an identity-related one.

Analysis - Sensory-mnemonic decisions. Exclusion criteria mirrored the main analysis. However, decisions were not excluded if we could not identify and match the search period preceding the first location-related decision since many behavioural sequences did not include a search at all (i.e., participants decided to re-encode from the Model after the first placement). Additionally, only decisions within sequences during which participants encoded with an object in their hand were included in this

supplementary analysis (30% of all sequences in 90° movement effort condition). Overall, 6308 decisions were included in the analysis for the first identity-related decision, 3359 decisions were included in analysis regarding the second location-related decisions, and 1712 decisions remained for the third identity-related decision.

The effect of distraction on the probability of using memory (i.e., sampling from Model vs. direct placement or pick-up using memory) was then investigated using GLMMs with a Binomial distribution. For all three decisions, we report outcomes from the full model. That is, the random effect structure included a subject intercept and by- subject random slopes distraction.

Analysis - Errors. Analyses for error rates were again based on the same data used to analyse the sensory-mnemonic decisions above and additional inclusion criteria mirrored the analyses presented in the main manuscript. The effect of distraction on location or identity errors (i.e., correct vs. incorrect placement or pick-up) was then investigated using GLMMs with a Binomial distribution. Separate models were run for the first and second pick-up or placement in the behavioural sequences respectively. For location errors at the first placement decisions, we report outcomes from the full model. That is, the random effect structure included a subject intercept and by- subject random slopes distraction. The random effect structure for the best fitting models for identity errors and location errors at the second placemen only included a subject intercept.

Results. To anticipate, these supplementary analyses corroborate the analyses reported in the main text. Specifically, identity-related decisions were again affected by our distraction manipulation, while location-related decisions seemed robust. Participants were less likely to pick up a second (β = -0.14, *SE* = 0.05, *z* =-2.65, *p* = 0.008, $CI_{95\%}$ = [-0.24,-0.04], BF_{10}= 0.84*; Supplementary Figure 5A*) and third object (β = -0.42, SE = 0.11, z =-3.99, p < 0.001, $CI_{95\%}$ = [-0.63,-0.21], BF_{10}= 9.00; *Supplementary Figure 5C*) after placing the already grabbed object, which would have required behaviour to be guided by identity attributes from memory.

When placing the already picked-up object, however, participants were not less likely to use memory when distraction was high (β = 0.23, SE = 0.11, z = 0.22, p = 0.83, $CI_{95\%}$ = [-0.18,0.23]; *Supplementary Figure 5B*). A follow-up Bayesian *t*-test (BF_{10}= 0.22) provided moderate evidence for the null hypothesis, indicating that the probability to place the object using its encoded location was not negatively affected by distraction.

Contrary to the main analyses, we do not find significantly more identity errors when picking-up a second object (β = -0.20, SE = 0.11, z =-1.80, p = 0.07, $CI_{95\%}$ = [-0.41,0.02], *Supplementary Figure 5E*). A follow-up Bayesian *t*-test (BF_{10}= 1.31) further provided no conclusive evidence for the effect. High distraction again did not lead to more location errors when placing the first (i.e., grabbed while encoding, β = 0.03, SE = 0.10, z = 0.31, p = 0.76, $CI_{95\%}$ = [-0.17,0.24]; *Supplementary Figure 5D*) or second (β = 0.04, SE = 0.14, z = 0.30, p = 0.76, $CI_{95\%}$ = [-0.21,0.29]; *Supplementary Figure 5F*) object. Follow-up Bayesian *t*-test provided moderate evidence for the null hypothesis for both the first (BF_{10}= 0.22) and second (BF_{10}= 0.22) placement, indicating no detrimental effect of distraction when relying on previously encoded location information.

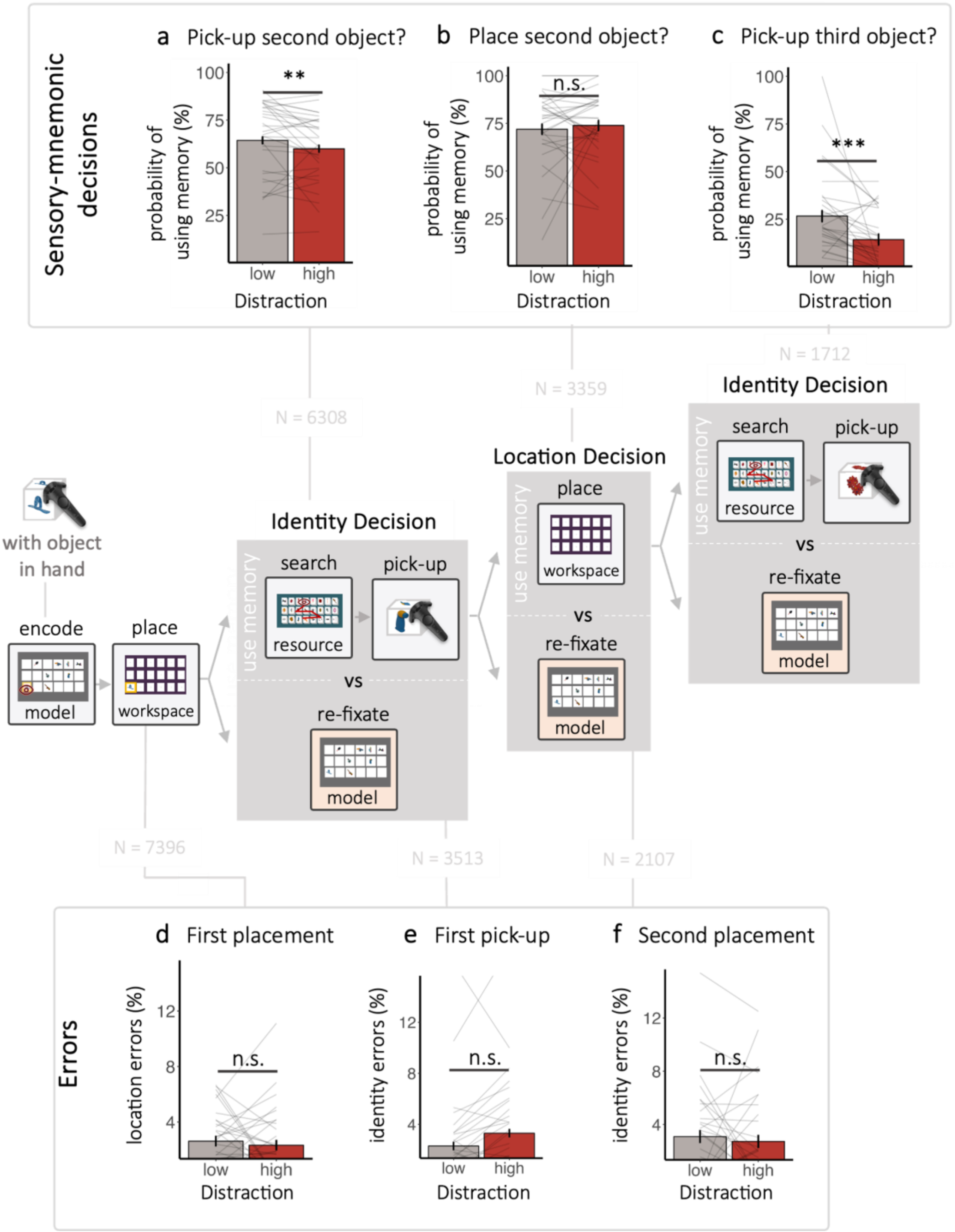

Supplementary Figure 5. *Influences of distraction on sensorimotor decisions and errors in "placement-first" sequences.* **a)** Placement of the first object is followed by an identity-related decision (i.e., Pick-up object using memory or re-fixate Model?) **b)** If participants place the object using memory, pick-up is followed by a location-related decision (i.e., Place a second object or re-fixate Model?) **c)** If memory was used again, a second identity-based decision would follow. **d)** Location errors while placing the initially grabbed object. **e)** Identity errors while picking up a second object, and **f)** location errors while placing a second object. All plots and analyses are based on the high movement effort condition. Error bars depict standard error of the mean (*N* = 30). Lines show individual participant data. N shows the number of sensory-mnemonic decisions or pick-ups/ placements included in the analyses. The symbols *, **, and *** in the figure denote statistical significance with p-values less than 0.05, 0.01, and 0.001, respectively.

Supplementary Table 2

Outcomes of LMMs analysing the effect of movement effort, distraction and their interaction on Display completion time and Total head movement.

Measure	Predictor	*ß*	*Std. Error*	*df*	t	p	η_p^2	95% CI
Display completion time	Movement effort	-0.084	0.003	29.120	-24.79	< 0.001	0.95	0.92, 0.97
	Distraction	0.054	0.003	28.548	18.23	< 0.001	0.92	0.86, 0.95
	Interaction	0.008	0.002	28.927	4.00	< 0.001	0.36	0.10, 0.57
Total head movement	Movement effort	-0.06	0.003	29.103	-22.071	< 0.001	0.94	0.90, 0.96
	Distraction	0.019	0.002	28.565	9. 145	< 0.001	0.75	0.56, 0.84
	Interaction	-0.001	0.002	29.195	-0.334	0.74	0.004	0.00, 0.14

Supplementary Table 3

Planned pairwise comparison breaking down the significant interaction between movement effort and distraction for Display completion time. P-values are Tukey-adjusted.

Measure	Comparison	*ß*	*Std. Error*	Z ratio	P_{corr}	Cohen's d	95% CI	BF_{10}
Display completion time	0°: high vs low distraction	0.125	0.008	15.907	< 0.001	-2.88	-3.70, -2.06	> 100
	90°: high vs low distraction	0.092	0.007	14.076	< 0.001	-2.59	-0.34, -1.83	> 100
	Low distraction: 0° vs 90	-0.182	0.008	-21.753	< 0.001	-3.98	-5.06, -2.90	> 100
	High distraction: 0° vs 90°	-0.149	0.007	-20.497	< 0.001	-3.76	-4.78, -2.73	> 100
	0°/low distraction vs 90°/high distraction	0.275	0.008	33.843	< 0.001	-6.20	-7.78, -4.57	> 100
	90°/low distraction vs 0°/high distraction	-0.057	0.010	-5.847	< 0.001	-1.07	-1.51, -0.61	> 100

.

Supplementary Table 4

Outcomes of LMMs and GLMMs analysing the effect of movement effort, distraction, and their interaction on measures for the encoding subcomponent (i.e., Total encoding time, Model viewing time, Model viewings per display, Number of targets encoded).

Measure	Predictor	*ß*	Std. Error	df	t/z	p	η_p^2	95% CI
Total encoding time	Movement effort	0.278	0.054	29.010	5.195	< 0.001	0.48	0.21, 0.66
	Distraction	0.139	0.028	28.652	5.035	< 0.001	0.47	0.20, 0.65
	Interaction	0.010	0.024	28.879	0.420	0.677	0.006	0.00, 0.16
Model viewing time	Movement effort	- 0.191	0.017	28.912	-11.437	< 0.001	0.82	0.68, 0.89
	Distraction	0.006	0.003	29.031	1.896	0.068	0.11	0.00, 0.34
	Interaction	0.006	0.003	27.316	2.339	0.027	0.17	0.01, 0.41
Model viewings per display	Movement effort	0.254	0.017	-	15.368	< 0.001	-	0.22, 0.29
	Distraction	0.030	0.006	-	5.186	< 0.001	-	0.02, 0.04
	Interaction	-0.009	0.006	-	-1.479	0.139	-	-0.02, 0.002
Number of targets encoded	Movement effort	-0.161	0.015	-	-10.974	< 0.001	-	-0.19,-0.13
	Distraction	0.006	0.004	-	1.614	0.107	-	-0.001, 0.01
	Interaction	0.003	0.004	-	0.693	0.488	-	-0.01, 0.01

Supplementary Table 5

Planned pairwise comparison breaking down the significant interaction between movement effort and distraction for Model viewing time. P-values are Tukey-adjusted.

Measure	Comparison	ß	*Std. Error*	Z ratio	P_{corr}	Cohen's *d*	95% CI	BF_{10}
Model viewing time	0°: high vs low distraction	0.0249	0.009	2.856	0.022	-0.52	-0.89, -0.13	5.19
	90°: high vs low distraction	< 0.0001	0.008	-0.001	1	-0.02	-0.38, 0.34	0.20
	Low distraction: 0° vs 90	-0.395	-0.395	-0.395	< 0.001	-2.09	-2.72, -1.44	> 100
	High distraction: 0° vs 90°	-0.370	0.033	-11.095	< 0.001	-2.03	-2.65, -1.39	> 100
	0°/low distraction vs 90°/high distraction	0.395	0.034	11.488	< 0.001	-2.10	-2.74, -1.45	> 100
	90°/low distraction vs 0°/high distraction	-0.370	0.034	-10.954	< 0.001	-1.99	-2.61 ,-1.36	> 100

Supplementary Table 6

Outcomes of LMMs and GLMMs analysing the effect of movement effort, distraction, and their interaction on measures for the visual search subcomponent (i.e., Search time, Number of targets/distractors looked at, target/distractor viewing times). *(*) model failed to converge.*

Measure	Predictor	*ß*	Std. Error	df	t/z	p	η_p^2	95% CI
Search time	Movement effort	0.0003	0.004	29.22	0.090	0.929	0.0002	0,0.07
	Distraction	0.098	0.004	28.95	26.159	< 0.001	0.96	0.93, 0.97
	Interaction	0.006	0.003	28.97	2.322	0.026	0.16	0.01, 0.40
Looked at targets (N)	Movement effort	-0.001	0.005	-	-0.182	0.85534	-	-0.01, 0.01
	Distraction	-0.022	0.007	-	-2.957	0.003	-	-0.04, -0.01
	Interaction	0.004	0.004	-	1.015	0.31025	-	-0.003, 0.01
Looked at distractors (N)	Movement effort	0.005	0.007	-	0.657	0.511	-	-0.01, 0.02
	Distraction	0.289	0.009	-	33.763	< 0.001	-	0.27, 0.31
	Interaction	- 0.007	0.005	-	- 1.368	0.171	-	-0.02, 0.003
Target viewing times (*)	Movement effort	-0.012	0.003	38320	-4.326	< 0.001	<0.001	0,0
	Distraction	0.017	0.006	28.49	2.939	0.006	0.23	0.02, 0.47
	Interaction	0.009	0.004	29.02	2.235	0.033	0.15	0, 0.38
Distractor viewing times	Movement effort	0.004	0.003	28.49	1.277	0.212	0.05	0, 0.27
	Distraction	0.092	0.003	29.40	31.123	< 0.001	0.97	0.95, 0.98
	Interaction	0.001	0.003	28.60	0.239	0.812	0.002	0, 0.11

Supplementary Table 7

Planned pairwise comparison breaking down the significant interaction between movement effort and distraction for Search time and Target viewing times. P-values are Tukey-adjusted.

Measure	Comparison	*ß*	*Std. Error*	Z ratio	P_{corr}	Cohen's *d*	95% CI	BF_{10}
Search time	0°: high vs low distraction	0.209	0.009	22.938	< 0.001	-4.20	-5.33, -3.06	> 100
	90°: high vs low distraction	0.185	0.009	20.517	< 0.001	-3.74	-4.76, -2.71	> 100
	Low distraction: 0° vs 90	- 0.011	0.009	-1.216	0.617	-0.22	-0.58, 0.14	0.38
	High distraction: 0° vs 90°	0.012	0.009	1.440	0.474	0.26	-0.11. 0.62	0.48
	0°/low distraction vs 90°/high distraction	0.196	0.010	19.962	< 0.001	-3.66	-4.66, -2.65	> 100
	90°/low distraction vs 0°/high distraction	0.198	0.011	17.876	< 0.001	3.27	2.35, 4.17	> 100
Target viewing times	0°: high vs low distraction	0.052	0.016	3.215	< 0.01	-0.56	-0.97, -0.19	11.31
	90°: high vs low distraction	0.015	0.012	1.330	0.544	-0.24	-0.60, 0.13	0.42
	Low distraction: 0° vs 90	- 0.043	0.010	-4.337	< 0.001	-0.67	-1.06, -0.27	34.93
	High distraction: 0° vs 90°	-0.006	0.010	-0.628	0.923	-0.12	-0.48, 0.24	0.24
	0°/low distraction vs 90°/high distraction	0.058	0.013	4.551	< 0.001	-0.74	-1.14, -0.33	86.19
	90°/low distraction vs 0°/high distraction	0.009	0.013	0.718	0.890	0.12	-0.24, 0.48	0.24

Supplementary Table 8

GLMMs analysing the effect of movement effort, distraction, and their interaction on measures on WM usage (i.e., Attributes used in memory). Note that in the second model, we included model viewing time as a predictor.

Measure	Predictor	*ß*	*Std. Error*	*df*	z	p	η_p^2	95% CI
Attributes used in WM (standard model)	Movement effort	-0.207	0.011	-	-18.166	< 0.001	-	-0.23,-0.18
	Distraction	-0.027	0.004	-	-7.023	< 0.001	-	-0.03,-0.01
	Interaction	0.007	0.003	-	2.232	0.0256	-	0.0001, 0.01
Attributes used in WM (predicted by encoding)	Movement effort	-0.183	0.010	-	-18.037	< 0.001	-	-0.20,-1.16
	Distraction	-0.027	0.004	-	-6.874	< 0.001	-	-0.03,-0.02
	Model viewing time	0.052	0.003	-	15.151	< 0.001	-	0.05, 0.06
	Interaction	0.007	0.003	-	1.992	0.0464	-	0.0001, 0.01

Supplementary Table 9

Planned pairwise comparison breaking down the significant interaction between movement effort and distraction for attributes used in WM for both the standard model and when predicted by encoding. P-values are Tukey-adjusted.

Measure	Comparison	*ß*	*Std. Error*	Z ratio	P_{corr}	Cohen's d	95% CI	BF_{10}
Attributes used in WM (standard model)	0°: high vs low distraction	-0.039	0.010	-3.924	< 0.001	0.76	0.35, 1.17	> 100
	90°: high vs low distraction	-0.068	0.010	-6.641	< 0.001	0.87	0.44, 1.29	> 100
	Low distraction: 0° vs 90	-0.429	0.024	-18.065	< 0.001	-2.38	-3.08,-1.67	> 100
	High distraction: 0° vs 90°	-0.340	0.024	-16.845	< 0.001	-2.55	-3.28,-1.80	> 100
	0°/low distraction vs 90°/high distraction	0.361	0.026	14.051	< 0.001	-2.14	-2.79,-1.48	> 100
	90°/low distraction vs 0°/high distraction	-0.468	0.022	-21.012	< 0.001	-2.66	-3.43,-1.89	> 100
Attributes used in WM (predicted by encoding)	0°: high vs low distraction	-0.041	0.010	-4.082	< 0.001	-	-	-
	90°: high vs low distraction	-0.067	0.011	-6.438	< 0.001	-	-	-
	Low distraction: 0° vs 90	-0.380	0.021	-17.751	< 0.001	-	-	-
	High distraction: 0° vs 90°	-0.353	0.021	-16.570	< 0.001	-	-	-
	0°/low distraction vs 90°/high distraction	0.312	0.024	13.174	< 0.001	-	-	-
	90°/low distraction vs 0°/high distraction	-0.421	0.020	-21.347	< 0.001	-	-	-

Supplementary Table 10

Pairwise comparisons comparing the probability of using different number of attributes in WM between distraction conditions, nested in movement effort conditions.

Measure	Comparison	*t*	*df*	p	Mean diff	Cohen's *d*	95% CI	BF_{10}
Probability (0° movement effort)	1 attribute: high vs low distraction	-2.19	29	0.04	-1.99	-0.4	-0.77,-0.02	1.54
	2 attributes: high vs low distraction	1.48	29	0.15	1.44	0.27	-0.1, 0.63	0.52
	3 attributes: high vs low distraction	1.37	29	0.18	0.47	0.25	-0.12, 0.61	0.45
	4 attributes: high vs low distraction	2.9	29	< 0.01	0.40	0.53	0.14, 0.91	6.15
Probability (90° movement effort)	1 attribute: high vs low distraction	-3.22	29	< 0.01	-2.50	-0.59	-0.97,-0.2	12.19
	2 attributes: high vs low distraction	-1.15	29	0.13	-2.26	-0.28	-0.65, 0.08	0.57
	3 attributes: high vs low distraction	2.56	29	0.02	1.81	0.47	0.09, 0.84	3.06
	4 attributes: high vs low distraction	2.94	29	< 0.01	2.08	0.54	0.15, 0.92	6.66

Supplementary Table 11

GLMMs analysing the effect of distraction on the different sensory-mnemonic decisions (i.e., both location- and identity-related decisions).

**Measure:** Probability of using memory	Predictor	*ß*	*Std. Error*	z	p	95% CI	BF_{10}
Decision 1: location-related	Distraction	-0.045	0.032	-1.394	0.163	-0.11, 0.02	0.38
Decision 2: identity-related	Distraction	-0.225	0.053	- 4.234	< 0.001	-0.33, -0.12	71.16
Decision 3: location-related	Distraction	0.238	0.076	3.12	0.002	0.09, 0.39	3.14

Supplementary Table 12

GLMMs analysing the effect of distraction on identity and location errors.

**Measure:**	Predictor	*ß*	*Std. Error*	z	p	95% CI	BF_{10}
Identity errors (first pick-up)	Distraction	0.273	0.075	3.636	< 0.001	0.13, 0.42	5.63
Location errors (first placement)	Distraction	-0.049	0.047	-1.039	0.299	-0.14, 0.04	0.45
Identity errors (second pick-up)	Distraction	0.358	0.102	3.506	< 0.001	0.16, 0.56	0.31
Location errors (second placement)	Distraction	0.320	0.135	2.368	0.0179	0.06, 0.59	2.16
