# Supplementary material for: Multifaceted consequences of visual distraction during natural behaviour
Source: Commun Psychol. 2024 May 27;2:49. doi: 10.1038/s44271-024-00099-0 (PMC11129948; doi:10.1038/s44271-024-00099-0)
Supplement: Supplementary file 3 — Description of Additional Supplementary Files [file 44271_2024_99_MOESM3_ESM.pdf]

Description of Additional Supplementary Files

File name: Supplementary Movie 1.

File Description: Illustration of the subcomponents within the VR Object copying task. The movie shows a trial in the high distraction and low movement effort condition. The red dot illustrates the participant's gaze, which was not visible to the participant during the task.

File name: Supplementary Movie 2.

File Description: Example trial in the low distraction condition. Visual distraction was manipulated by changing the opacity of the distractor objects in the resource pool. The red dot illustrates the participant's gaze, which was not visible to the participant during the task.

File name: Supplementary Movie 3.

File Description: Example trial in the high movement effort condition. Movement effort was manipulated by rotating the model display 90° away from the workspace. The red dot illustrates the participant's gaze, which was not visible to the participant during the task.
