# Supplementary material for: Multifaceted consequences of visual distraction during natural behaviour
Source: Commun Psychol. 2024 May 27;2:49. doi: 10.1038/s44271-024-00099-0 (PMC11129948; doi:10.1038/s44271-024-00099-0)
Supplement: Supplementary file 7 — Reporting Summary [file 44271_2024_99_MOESM7_ESM.pdf]

nature portfolio

Corresponding author(s): Levi Kumle

Last updated by author(s): Apr 24, 2024

Reporting Summary

Nature Portfolio wishes to improve the reproducibility of the work that we publish. This form provides structure for consistency and transparency in reporting. For further information on Nature Portfolio policies, see our Editorial Policies and the Editorial Policy Checklist.

Statistics

For all statistical analyses, confirm that the following items are present in the figure legend, table legend, main text, or Methods section.

n/a	Confirmed	
☐	☒	The exact sample size (*n*) for each experimental group/condition, given as a discrete number and unit of measurement
☐	☒	A statement on whether measurements were taken from distinct samples or whether the same sample was measured repeatedly
☐	☒	The statistical test(s) used AND whether they are one- or two-sided<br>*Only common tests should be described solely by name; describe more complex techniques in the Methods section.*
☒	☐	A description of all covariates tested
☐	☒	A description of any assumptions or corrections, such as tests of normality and adjustment for multiple comparisons
☐	☒	A full description of the statistical parameters including central tendency (e.g. means) or other basic estimates (e.g. regression coefficient) AND variation (e.g. standard deviation) or associated estimates of uncertainty (e.g. confidence intervals)
☐	☒	For null hypothesis testing, the test statistic (e.g. *F*, *t*, *r*) with confidence intervals, effect sizes, degrees of freedom and *P* value noted<br>*Give P values as exact values whenever suitable.*
☐	☒	For Bayesian analysis, information on the choice of priors and Markov chain Monte Carlo settings
☒	☐	For hierarchical and complex designs, identification of the appropriate level for tests and full reporting of outcomes
☐	☒	Estimates of effect sizes (e.g. Cohen's *d*, Pearson's *r*), indicating how they were calculated

Our web collection on statistics for biologists contains articles on many of the points above.

Software and code

Policy information about availability of computer code

Data collection	The virtual environment and experiment were programmed and run in Unity (version 2019.3; Unity Technologies) using the SteamVR Unity plugin (version 1.2.10; Valve Corporation) on a computer operated with Windows 10.
Data analysis	All data was pre-processed and analysed in the R statistical programming language (version 4.1.2; R Core Team, 2021) using RStudio (version 2.3.492; RStudio Team, 2022).

For manuscripts utilizing custom algorithms or software that are central to the research but not yet described in published literature, software must be made available to editors and reviewers. We strongly encourage code deposition in a community repository (e.g. GitHub). See the Nature Portfolio guidelines for submitting code & software for further information.

Data

Policy information about availability of data

All manuscripts must include a data availability statement. This statement should provide the following information, where applicable:

- Accession codes, unique identifiers, or web links for publicly available datasets
- A description of any restrictions on data availability
- For clinical datasets or third party data, please ensure that the statement adheres to our policy

The post-processed data (segmented and summarised) are available at the Open Science Framework: https://osf.io/ze5p3/?view_only=c93d737d187f4084bfc425b71b8c5b3c

Raw data is available from the authors upon request.

Human research participants

Policy information about studies involving human research participants and Sex and Gender in Research.

Reporting on sex and gender	Sex and gender were not considered in the study design. Participants self-reported gender identity (22 women, 8 men, 0 other).
Population characteristics	Mean age = 24.5, range = 18-32, 26 right-handed, 4 left-handed, all self-reported, normal or corrected-to-normal vision (contact lenses; including colour vision)
Recruitment	Recruitment at random
Ethics oversight	The research protocol was approved by the local ethics committee of the Faculty of Psychology and Sport Sciences at Goethe University Frankfurt as well as the Central University Research Ethics Committee, University of Oxford (#R64089/RE001).

Note that full information on the approval of the study protocol must also be provided in the manuscript.

Field-specific reporting

Please select the one below that is the best fit for your research. If you are not sure, read the appropriate sections before making your selection.

☐ Life sciences ☒ Behavioural & social sciences ☐ Ecological, evolutionary & environmental sciences

For a reference copy of the document with all sections, see nature.com/documents/nr-reporting-summary-flat.pdf

Behavioural & social sciences study design

All studies must disclose on these points even when the disclosure is negative.

Study description	We ran an experimental virtual reality study.
Research sample	Thirty participants (Mean age = 24.5, range = 18-32, 22 women, 8 men, 0 other, 26 right-handed, 4 left-handed, all self-reported) were recruited at Goethe University Frankfurt (n = 20) and the University of Oxford (n = 10). All participants had normal or corrected-to-normal vision (contact lenses; including colour vision) and provided informed consent prior to participating in the study.
Sampling strategy	Recruitment was at random or through convenience. Sample size planning was guided by a previous study using a similar VR paradigm (Draschkow et al. 2021), combined with a high trial-number approach to maximise the number of observations per participant. We ran simulation-based power analyses for the outcome variables attributes used in memory, display completion time and model viewing time based on data from Draschkow et al. (2021). We explored power over a range of simulated effect sizes for a main effect of distraction (for details see shared materials). Thirty participants, each completing 224 trials, yielded power > 90% to detect an absolute mean difference of 0.04 attributes used in memory, 0.7 seconds in display completion time, and 8 milliseconds in model viewing time between distraction conditions.
Data collection	Participants were equipped with an HTC Vive Tobii Pro VR integration with a built-in binocular eye tracker and one wireless HTC Vive Controller in their dominant hand. Frame-by-frame (90 Hz) data was written into csv-files during recording. For each frame, we recorded which objects in the VR environment were looked at, location in space of both HMD and controller as well as all relevant interactions with the environment (e.g., if and which object was grabbed).
Timing	May - June 2021
Data exclusions	Only trials that were completed before the timeout (i.e., < 45 seconds) were included in the analyses of overall behaviour (101 trials excluded; 1.5% of data). Additional exclusion criteria apply to analysis on the separate sub-components of behaviour. These are clearly stated in the main manuscript.
Non-participation	In total, one participant did not complete the second testing day without stating a reason.
Randomization	Experimental conditions were manipulated within-participant.

Reporting for specific materials, systems and methods

We require information from authors about some types of materials, experimental systems and methods used in many studies. Here, indicate whether each material, system or method listed is relevant to your study. If you are not sure if a list item applies to your research, read the appropriate section before selecting a response.

Materials & experimental systems

n/a	Involved in the study
☒	☐ Antibodies
☒	☐ Eukaryotic cell lines
☒	☐ Palaeontology and archaeology
☒	☐ Animals and other organisms
☒	☐ Clinical data
☒	☐ Dual use research of concern

Methods

n/a	Involved in the study
☒	☐ ChIP-seq
☒	☐ Flow cytometry
☒	☐ MRI-based neuroimaging
